# Restoration of bone mineral density in a patient with congenital adrenal hyperplasia using hydrocortisone pump therapy

**DOI:** 10.1210/jcemcr/luag036

**Published:** 2026-04-02

**Authors:** Rebecca Gorrigan, Benjamin Todd, John W Honour, Peter C Hindmarsh

**Affiliations:** Department of Endocrinology, St Bartholomew's Hospital, London EC1A 7BE, UK; The Harvey Practice Broadstone, Poole BH18 8EE, UK; Institute for Women's Health, University College London, London WC1E 6BT, UK; Genomics and Genetic Medicine, University College London, London WC1N 1EH, UK

**Keywords:** bone density, DXA, glucocorticoids, osteoporosis, hydrocortisone pump

## Abstract

Osteoporosis is a major public health problem. Glucocorticoids alter the number of cells within bone, reducing bone forming cells (osteoblasts) and those that resorb bone (osteoclasts), the former more so than the latter. Glucocorticoid-induced osteoporosis predominately affects regions of the skeleton such as the spine and the neck of the femur. We describe a 14.5-year-old male with congenital adrenal hyperplasia who developed osteopenia (spinal bone mineral density [BMD] *Z*-score −1.5; hip BMD −1.7; previously −0.2 and −1.7, respectively) as a result of treatment with prednisolone and high doses of dexamethasone. He was subsequently shown to have a rapid clearance and a half-life of 40 minutes (average normal value 80 minutes) for hydrocortisone. Hydrocortisone delivery using a continuous tiered subcutaneous infusion pump to mimic the normal cortisol circadian rhythm normalized all biochemical parameters and prevented further bone mineral loss over the following 5 years and over 20 years of treatment normalized BMD (spine *Z*-score +0.4; hip *Z*-score +0.7). Continuous tiered subcutaneous hydrocortisone pump therapy delivering plasma cortisol concentrations indistinguishable from normal individuals, can prevent bone mineral loss and over a long period leads to a normalization of BMD.

## Introduction

Patients with congenital adrenal hyperplasia (CAH) receive lifelong glucocorticoid replacement therapy, which brings with it potential long-term bone problems such as reduced bone mineral density (BMD), osteoporosis, and an increased risk of fracture [1]. These observations relate to high doses of high-potency glucocorticoids used for their anti-inflammatory properties and not replacement therapy. The extent of the problem with BMD in individuals with CAH due to 21-hydroxylase enzyme deficiency (hereafter referred to as CAH) shows a mixed picture with some studies showing normal [2] or high [3] BMD, whereas a meta-analysis comparing patients with CAH and matched controls found a slightly decreased BMD in CAH patients [4]. These variations may reflect differences in glucocorticoid dosing schedules, the use of potent glucocorticoids such as dexamethasone [1, 5], age at diagnosis, and overall exposure to adrenal androgens. The latter, resulting from poor replacement therapy or a later diagnosis, may improve BMD because of high androgen concentrations [6].

Glucocorticoid replacement therapy needs to be individualized because of the marked variation between individuals in terms of absorption and clearance [7]. When biochemical control is viewed as suboptimal, common practice is to increase glucocorticoid dosing or switch to more potent glucocorticoids. We previously described a 14.5-year-old boy, in whom hydrocortisone pharmacokinetics were altered with a short half-life of 50 and 40 minutes on retesting 5 and 10 years later, who was administered prednisolone and dexamethasone therapy to achieve androgen control, causing gastrointestinal side effects, and in whom long-term control has been achieved for more than 20 years using a continuous tiered subcutaneous infusion of hydrocortisone (CSIH) [8]. Prednisolone and dexamethasone had an adverse effect on BMD, which was reversed and normalized over this period with CSIH.

## Case presentation

The full details of the case at instigation of CSIH treatment have been previously reported [8]. In brief, the patient was 14.5 years of age with consistently high plasma 17-hydroxyprogesterone (17OHP) and ACTH concentrations, virilization without testicular progression through puberty, and recurrent headaches.

## Diagnostic assessment

Biochemical assessment at the age of 14.5 years revealed plasma ACTH 350 pg/mL (reference range 10-50 pg/mL), plasma 17OHP 164 ng/mL (Systeme International [SI]: 497 nmol/L) (reference range, 0.3-1.7 ng/mL [SI: 1-5 nmol/L]), serum testosterone 7.6 ng/mL (SI: 26.5 nmol/L) (reference range, 3.8-8.1 ng/mL [SI: 13-28 nmol/L]), and serum androstenedione 4.8 ng/mL (SI: 16.8 nmol/L) (reference range, 0.3-2.9 ng/mL [SI: 1-10 nmol/L]) despite reasonable doses of hydrocortisone. The testosterone is relatively normal despite the high ACTH and 17OHP levels because the conversion of androstenedione to testosterone in the adrenal glands is not very effective.

Prednisolone therapy (9 mg/m^2^ body surface area/day) for 3 months commencing in July 2004 did not alter the serum 17OHP concentration, which averaged 24.1 ng/mL (SI: 73 nmol/L) at 8:00 Am and was discontinued because of gastrointestinal side effects and rapid weight gain, as was dexamethasone after 1 month of therapy. Over two 48-hour periods of dexamethasone, the plasma ACTH after 48 hours of dexamethasone therapy (1 mg/m^2^ body surface area/day given as 2 equal doses) followed by 48 hours of 4 mg/m^2^ body surface area/day were <5.0 pg/mL respectively. Plasma 17OHP was 175 ng/mL (SI: 530 nmol/L) at the start of the dexamethasone, 90 ng/mL (273 nmol/L) after the first 48 hours of dexamethasone, and 1.7 ng/mL (SI: 5.2 nmol/L) after the higher dose of dexamethasone given for a further 48 hours.

Pharmacokinetic studies revealed a normal volume of distribution and reduced half-life of 50 minutes (normal range 70-100 minutes) and 40 minutes when retested 5 and 10 years later. The bioavailability of 30 mg of hydrocortisone given by the oral and intramuscular routes was 42% (normal 90% and 100%, respectively). Subsequent studies demonstrated a marked increase in urinary cortisone metabolites compared to urinary cortisol metabolites, indicating increased conversion of cortisol to cortisone by 11β-hydroxysteroid dehydrogenase type 2.

Bone mineral density assessment by dual photon absorption (DXA) (Hologic Horizon, Manchester, UK) scanning was undertaken before treatment with prednisolone and dexamethasone, 6 months following prednisolone and dexamethasone treatment, and at 12- to 24-month intervals apart from 2019 to 2023, where follow up was disrupted due to COVID-19 and transition to adult services. There are a variety of ways of recording the data either as a *t*-score, which show the individual's DXA results compared to the ideal peak bone density of a healthy adult, or as a *Z*-score, which compares the individual's DXA results to the average reference range based on the same age, weight, height, and gender of the general population as the individual. We chose the latter to allow for easier comparisons to the general population over time.

## Treatment

To overcome the bioavailability and clearance problems, treatment with CSIH was commenced initially using a Medtronic MiniMed 712 Insulin Infusion device and more recently the 640G version (Medtronic Diabetes, Watford, UK) with hydrocortisone as Solu-cortef (Pfizer Pharmaceuticals, Sandwich, UK) in a concentration of 100 mg/mL. The basal infusion rate was set at 5 different delivery rates over different time periods through the 24-hour cycle using the patient's hydrocortisone pharmacokinetics to deliver a cortisol circadian rhythm.

## Outcome and follow-up

Plasma ACTH (5.0 pg/mL) and serum androstenedione (0.8 ng/mL [SI: 2.8 nmol/L]) concentrations were rapidly returned to within the normal range within 3 months of commencing therapy and have remained at similar values over 20 years. Serum testosterone decreased initially from 4.7 ng/mL (SI: 16.4 nmol/L) before CSIH to 4.2 ng/mL (SI: 14.7 nmol/L) at 3 months rising to 5.8 ng/mL (SI: 20.0 nmol/L) at 2 years an increase coincident with an increase in testicular volume.

The pump rates reproduced the normal circadian rhythm of plasma cortisol that was indistinguishable from the rhythm observed in control subjects [9]. Plasma 17OHP concentrations measured over the 24-hour period were not suppressed but were within the normal range of 0.3 to 0.7 ng/mL (SI: 1-5 nmol/L).

Following the introduction of CSIH, symptoms of tiredness and headaches abated. Before commencing pump therapy, the patient was very underweight. With the introduction of CSIH, hydrocortisone doses were reduced from 80 mg/day (46 mg/m^2^/day) to 32 mg/day (18.5 mg/m^2^/day) by titration based on careful 24-hour plasma cortisol profiling. His weight returned to normal and has remained stable over the years with no weight gain. The patient was able to return to school, go to university, and holds down a very demanding and responsible managerial role. He partakes in sports, such as surfing and sea swimming, regularly attends gym, partakes in CrossFit competitions, and lives a very full active life. We initially used the hydrocortisone sodium phosphate preparation instead of Solu-cortef but this caused infusion site problems because he was allergic to this preparation.

Table 1 shows the changes in BMD expressed as absolute BMD and as *Z*-scores for the spine and hip during the period of follow up. Before the introduction of prednisolone (3 months of therapy at a dose of 9 mg/m^2^ body surface area/day) followed by dexamethasone (1 month of therapy at a dose of 2 mg/m^2^ body surface area/day) the spine BMD *Z*-score (−0.2) was normal probably reflecting the raised concentrations of adrenal androgens at that stage. Four months after cessation of dexamethasone, the BMD *Z*-score particularly at the spine had decreased to −1.1 and remained at this value over the next 2 years. Thereafter BMD *Z*-scores at the spine and hip sites improved slowly and by 2024 had normalized. Absolute BMD values changed in a similar manner to the *Z*-scores.

**Table 1:** Effect of treatment with continuous tiered subcutaneous infusion of hydrocortisone (CSIH) over 20 years on bone mineral density expressed as absolute bone mineral density and as *Z*-scores in an individual with congenital adrenal hyperplasia

Date	BMD spine (L1-L4) (g/cm^2^)	BMD spine (L1-L4) *Z*-score	BMD hip (average of left and right) (g/cm^2^)	BMD hip (average of left and right) *Z*-score	Comment
2004*^a^*	0.914 g/cm^2^	−0.2	0.781 g/cm^2^	−1.7	Pre-prednisolone and dexamethasone, which commenced in July 2004
2005*^a^*	0.853 g/cm^2^	−1.1	0.774 g/cm^2^	−1.8	4 months after cessation of prednisolone and dexamethasone (March 2005) and start of CSIH therapy in October 2005
2006*^a^*	0.919 g/cm^2^	−1.0	0.781 g/cm^2^	−1.7	
2009	0.924 g/cm^2^	−1.5	0.777 g/cm^2^	−1.7	
2011	0.967 g/cm^2^	−1.1	0.794 g/cm^2^	−1.6	
2016	0.950 g/cm^2^	−1.3	0.842 g/cm^2^	−1.2	
2017	0.970 g/cm^2^	−1.1	0.860 g/cm^2^	−1.0	
2024	1.297 g/cm^2^	0.4	0.989 g/cm^2^	0.7	

Abbreviation: BMD, bone mineral density

^*a*^Spinal bone mineral density adjusted for height during completion of puberty.

## Discussion

Conventional oral hydrocortisone dosing schedules do not mimic the normal cortisol circadian rhythm, which makes the control of CAH a balance between over- and underexposure to glucocorticoid. In this case report, BMD was normal at presentation, presumably because of the elevations in adrenal androgen production [6]. The use of high-potency glucocorticoids resulted in a marked loss of BMD, which particularly affected the spine. This is consistent with the observation that vertebral fractures are the most common fractures associated with high-dose glucocorticoid use. The risk of a vertebral fracture increases within 3 months after initiation of treatment and peaks at 12 months. The relative risk of a fracture doubles and the risk of hip fracture increase by 50% among people receiving 2.5 to 7.5 mg of prednisolone daily (doses not too far from replacement therapy, reflecting the greater potency of prednisolone compared to hydrocortisone). Similar risk statistics apply at the spine and hip sites with a clear increase once total daily prednisolone doses exceed 2.5 mg per day [1].

The daily dose of hydrocortisone administered by CSIH was higher than that used for the management of Addison disease using CSIH, reflecting the higher dose of glucocorticoid needed to suppress adrenal androgen production in CAH along with the altered hydrocortisone kinetics in this individual. Nonetheless, the current dose is approximately 60% of that given by the oral route, which was also associated with poor CAH control. This emphasizes the need to individualize glucocorticoid replacement therapy, regardless of the mode of administration in individuals with adrenal insufficiency, due to interindividual variations in absorption and clearance [7].

Continuous tiered subcutaneous infusion of hydrocortisone has been used in several patients with adrenal insufficiency. In CAH short-term studies (3-18 months) have confirmed the efficacy of this approach in several biochemical parameters, but no changes have been noted to BMD [10, 11]. This may reflect the short-term nature of follow up as changes in BMD can take from several months to 2 to 3 years to manifest. This would appear to be the situation with our patient, where there was the classic very rapid loss of BMD upon initiation of treatment with the high-potency glucocorticoids, which would have been coupled with the loss of the anabolic effects of the adrenal androgens on bone. The most affected area, as expected, was the spine and normalization of plasma cortisol concentrations with CSIH prevented further bone mineral loss, allowing a gradual recovery in BMD [1, 12]. Unfortunately, we cannot say exactly when a normal BMD was attained because of problems around COVID-19 access to investigations. Recovery of a reduced BMD resulting from the use of high-dose and/or potency glucocorticoids is less well-documented in the literature, probably reflecting the need for repeated courses of glucocorticoids when used for their anti-inflammatory properties. This case report suggests that recovery can take several years.

In this individual, CSIH over a period of 20 years, has been effective in maintaining good health, quality of life, and in particular the gradual restoration of BMD that was originally lost by the use of high-potency glucocorticoids. This therapeutic approach has been used by several other groups and highlights the importance of approximating as closely as possible the normal circadian rhythm of cortisol. Closing the feedforward and feedback loop by physiological cortisol replacement normalizes the biochemical parameters taken as representative of androgen control in CAH without the use of high doses of glucocorticoid.

## Learning points

- Continuous tiered subcutaneous infusion of hydrocortisone reproduces exactly the normal circadian pattern of cortisol normalizing plasma 17-hydroxyprogesterone and androstenedione concentrations.
- The use of high doses of high-potency glucocorticoids even for short periods of 3 to 4 months leads to a loss of bone mineral density.
- Using CSIH prevented further loss of bone mineral density with a gradual restoration and normalization over a long period.

## Contributors

All authors made individual contributions to authorship. J.W.H. and P.C.H. identified the rapid clearance of hydrocortisone and instigated the management of hydrocortisone replacement pump therapy using continuous subcutaneous infusion of hydrocortisone. R.G. provided adult endocrine input and further monitoring. B.T. coordinated care. All authors reviewed and approved the final draft.

## Data Availability

Original data generated and analyzed during this study are included in this published article.

## References

[1] Van Staa TP, Leufkens HGM, Abenhaim L, Zhang B, Cooper C. Use of oral corticosteroids and risk of fractures. J Bone Miner Res. 2000;15(6):993‐1000. 10841167 10.1359/jbmr.2000.15.6.993

[2] Stikkelbroeck NM, Oyen WJ, van der Wilt GJ, Hermus AR, Otten BJ. Normal bone mineral density and lean body mass, but increased fat mass, in young adult patients with congenital adrenal hyperplasia. J Clin Endocrinol Metab. 2003;88(3):1036‐1042. 12629082 10.1210/jc.2002-021074

[3] Arisaka O, Hoshi M, Kanazawa S, et al Preliminary report: effect of adrenal androgen and estrogen on bone maturation and bone mineral density. Metabolism. 2001;50(4):377‐379. 11288028 10.1053/meta.2001.21678

[4] Rangaswamaiah S, Gangathimmaiah V, Nordenstrom A, Falhammar H. Bone mineral density in adults with congenital adrenal hyperplasia: a systematic review and meta-analysis. Front Endocrinol (Lausanne). 2020;11:493. 32903805 10.3389/fendo.2020.00493 PMC7438951

[5] Whittle E, Falhammar H. Glucocorticoid regimens in the treatment of congenital adrenal hyperplasia: a systematic review and meta-analysis. J Endocr Soc. 2019;3(6):1227‐1245. 31187081 10.1210/js.2019-00136 PMC6546346

[6] Falhammar H, Filipsson H, Holmdahl G, et al Fractures and bone mineral density in adult women with 21-hydroxylase deficiency. J Clin Endocrinol Metab. 2007;92(12):4643‐4649. 17878254 10.1210/jc.2007-0744

[7] Hindmarsh PC, Charmandari E. Variation in absorption and half-life of hydrocortisone influence plasma cortisol concentrations. Clin Endocrinol (Oxf). 2015;82(4):557‐561. 25369980 10.1111/cen.12653

[8] Bryan SM, Honour JW, Hindmarsh PC. Management of altered hydrocortisone pharmacokinetics in a boy with congenital adrenal hyperplasia using a continuous subcutaneous hydrocortisone infusion. J Clin Endocrinol Metab. 2009;94(9):3477‐3480. 19567522 10.1210/jc.2009-0630

[9] Hindmarsh PC, Geertsma K. Replacement Therapies in Adrenal Insufficiency. Chapter 11. Elsevier Inc; 2024.

[10] Merza Z, Rostami-Hodjegan A, Memmott A, et al Circadian hydrocortisone infusions in patients with adrenal insufficiency and congenital adrenal hyperplasia. Clin Endocrinol (Oxf). 2006;65(1):45‐50. 16817818 10.1111/j.1365-2265.2006.02544.x

[11] Mallappa A, Nella AA, Sinaii N, et al Long-term use of continuous subcutaneous hydrocortisone infusion therapy in patients with congenital adrenal hyperplasia. Clin Endocrinol (Oxf). 2018;89(4):399‐407. 30003563 10.1111/cen.13813 PMC6166869

[12] Laurent MR, Goemaere S, Verroken C, et al Prevention and treatment of glucocorticoid-induced osteoporosis in adults: consensus recommendations from the Belgian bone club. Front Endocrinol (Lausanne). 2022;13:908727. 35757436 10.3389/fendo.2022.908727 PMC9219603

